# Emotional Intelligence and Teacher Self-Efficacy in Initial Teacher Education: A Psychoeducational Intervention with Spanish Pre-Service Teachers

**DOI:** 10.3390/jintelligence14050075

**Published:** 2026-05-01

**Authors:** Lorena González-Ros, Teresa Pozo-Rico, Juan Luis Castejón, Raquel Gilar-Corbí

**Affiliations:** Department of Developmental Psychology and Teaching, Faculty of Education, University of Alicante, 03690 San Vicente del Raspeig, Alicante, Spain; lorena.gonzalezros@ua.es (L.G.-R.); jl.castejon@ua.es (J.L.C.); raquel.gilar@ua.es (R.G.-C.)

**Keywords:** emotional intelligence, self-efficacy, teacher training, psychoeducational intervention, emotional education

## Abstract

Emotional intelligence and teaching self-efficacy are essential competencies for teachers’ professional and personal development. The aim of this study was to evaluate the effectiveness of a psychoeducational intervention to enhance both areas in future teachers. A quasi-experimental design with pretest and posttest measures was implemented, including an experiment. An eight-week program was conducted using active, reflective, and participatory methodologies to promote emotional awareness and confidence in teaching abilities. The OSTES instrument was used to measure teaching self-efficacy, the TMMS-24 to assess perceived emotional intelligence, and the EQ-i to evaluate socioemotional competencies, and. Results revealed significant improvements in the experimental group in emotional attention, clarity, and repair; in instructional strategies, classroom management, and student engagement; as well as in adaptability, interpersonal skills, stress management, and overall emotional intelligence. These effects ranged from moderate to large in magnitude and contrasted with the stable scores in the control group. The findings confirm that psychoeducational interventions focused on emotional competencies can be effective in strengthening emotional intelligence and self-efficacy in pre-service teachers. These outcomes suggest that such programs may contribute to the promotion of well-being and teaching effectiveness during initial teacher education, offering implications for future curricular development in teacher training programs.

## 1. Introduction

In the context of today’s increasingly complex educational landscape—marked by classroom diversity, growing academic demands, and emotional challenges—the development of socio-emotional competencies and the strengthening of teacher self-efficacy have emerged as essential components of high-quality teacher training. Initial teacher education can no longer focus exclusively on pedagogical and disciplinary knowledge, but must also foster personal, emotional, and social skills that enable prospective teachers to navigate the demands of their profession with resilience, flexibility, and effectiveness (Procópio et al., 2023). Given the emotionally, socially, and cognitively demanding nature of teaching, pre-service teachers require preparation that integrates these competencies alongside content and pedagogy.

International organizations such as the OECD (2021) and UNESCO (2023) have highlighted the need to integrate socio-emotional learning as a core component in teacher education programs, and empirical evidence likewise indicates that emotional intelligence (EI) and perceived teacher self-efficacy are closely linked to teachers’ effectiveness and well-being (Jennings & Greenberg, 2009). Higher emotional intelligence has been associated with improved classroom climate management, more positive student-teacher relationships, and better coping with occupational stress (Barrientos et al., 2019). Likewise, teacher self-efficacy, the belief in one’s capacity to positively influence student learning, has been related to professional commitment, pedagogical innovation, perseverance, and teacher well-being (Gale et al., 2021; Zimmerman, 2000).

Despite this growing recognition, the recent literature points to a critical shortage of empirically validated socio-emotional intervention programs, particularly within initial teacher training and in Spanish-speaking countries. Specifically, three major gaps persist: (1) a lack of experimental or quasi-experimental designs assessing the effectiveness of socio-emotional interventions in pre-service teacher education; (2) a scarcity of studies that simultaneously evaluate emotional intelligence as a trait (EQ-i), perceived emotional intelligence (TMMS-24), and teacher self-efficacy (OSTES); and (3) limited evidence demonstrating pre-post group differences that allow the attribution of observed changes to the intervention itself, rather than to natural developmental processes or external influences (Torres-Hernández, 2025).

The present study addresses these gaps through the implementation of an eight-week psychoeducational program specifically designed for future Early Childhood and Primary Education teachers in Spain. The intervention is grounded in active, experiential, and reflective methodologies, aimed at enhancing emotional intelligence and teacher self-efficacy, and equipping future teachers with practical tools to manage classroom emotional climate, regulate stress, and promote student engagement. As supported by recent literature, initial teacher education presents a strategic opportunity to foster these competencies when approached through purposefully structured training programs (Zhi et al., 2024).

Therefore, implementing psychoeducational interventions that foster both emotional intelligence and perceived teaching self-efficacy emerges as a timely and necessary response aligned with current demands for a more human, reflective, and emotionally competent teaching profession.

This study aims to provide robust empirical evidence from the Spanish context, contributing to international discussion on best practices in initial teacher education. Specifically, it seeks to evaluate the effectiveness of a structured psychoeducational intervention, offering a replicable and scalable model for diverse educational systems. In doing so, the study responds to recent calls for more comparable evidence on the effectiveness of socio-emotional training in initial teacher education. The results may inform future curricular policies and promote the integration of socio-emotional learning into teacher education programs.

### 1.1. Theoretical Framework

#### 1.1.1. Emotional Intelligence in Educational Contexts

The concept of emotional intelligence (EI) has gained significant relevance in recent years, both in the field of psychology and education. Broadly defined, EI refers to the ability to accurately identify, understand, express, and regulate one’s own emotions as well as those of others (Mayer & Salovey, 1997). The literature has consistently demonstrated its impact on psychological well-being, the quality of interpersonal relationships, academic achievement, and professional performance in both organizational and educational settings (Goleman & Cherniss, 2024). However, most of the existing research has been based on correlational designs, making it difficult to determine whether improvements in EI through intervention programs can produce causal effects on perceived teacher self-efficacy.

Several theoretical approaches have conceptualized EI. The ability model, developed by Mayer and Salovey (1997), frames EI as a set of cognitive abilities organized into four hierarchical branches: emotional perception, emotional facilitation of thought, emotional understanding, and emotional regulation. The trait or perceived EI approach, introduced by Petrides and Furnham (2001) and later consolidated within the personality domain (Petrides et al., 2007), conceptualizes EI as emotional self-perceptions typically assessed using self-report instruments such as the TMMS-24. The mixed model proposed by Bar-On (1997, 2006), operationalized through the EQ-i, incorporates broader emotional and social competencies, offering a global profile of socio-emotional functioning.

In educational contexts, EI is particularly relevant for teachers, who continuously engage in emotionally charged interactions with students, colleagues, and families. The ability to manage stress, interpret emotional cues in the classroom, and respond empathetically and constructively has a direct impact on classroom climate, behavior management, and pedagogical effectiveness (Rauterkus et al., 2024; Taxer & Gross, 2018). Empirical research supports the positive influence of EI on professional development: higher levels of EI have been associated with reduced emotional exhaustion, greater job satisfaction, better classroom management, and more positive relationships with students (D’Amico et al., 2020; Extremera & Fernández-Berrocal, 2005; Cejudo & López-Delgado, 2017; Wang, 2023; Yin, 2015).

In the present study, both the TMMS-24 and the EQ-i were used, allowing for a more comprehensive and multidimensional approach to assessing perceived emotional intelligence and the socio-emotional competencies of future teachers.

#### 1.1.2. Teacher Self-Efficacy: Concept, Dimensions, and Educational Implications

Self-efficacy is a central concept in Bandura’s social cognitive theory, defined as an individual’s belief in their ability to organize and execute the actions required to manage prospective situations (Bandura, 1997, 2013; Ewen, 2014). In educational settings, perceived teacher self-efficacy refers to teachers’ beliefs about their capability to positively influence student learning, manage classroom behavior, and motivate students, even under challenging conditions (Tschannen-Moran & Hoy, 2001).

According to Bandura (1997), self-efficacy develops from four primary sources: (1) mastery experiences or previous achievements, (2) vicarious learning through observing others, (3) social or verbal persuasion (positive feedback), and (4) physiological and emotional regulation. In initial teacher education, these sources can be intentionally fostered through reflective practice, guided simulations, structured peer feedback, and supervised teaching experiences.

The OSTES model (Teachers’ Sense of Efficacy Scale) identifies three core dimensions of teacher self-efficacy: instructional strategies, classroom management, and student engagement. These dimensions encompass teachers’ capacity to design and implement effective teaching methods, handle disruptive behaviors, and motivate and engage students in the learning process (Tschannen-Moran & Hoy, 2001, 2007, 2012). These domains have been linked to key indicators of educational quality such as student-centered pedagogy, persistence in the face of adversity, pedagogical innovation, adaptability, and stress management (Holzberger et al., 2013; Kholifah et al., 2023; Künsting et al., 2016). Furthermore, high levels of self-efficacy act as a protective factor against burnout and attrition from the profession (Kim & Burić, 2020).

Due to its dynamic and malleable nature, self-efficacy can be developed during initial teacher education, facilitating a smoother and more confident transition into the teaching profession. However, despite the robust correlational evidence linking self-efficacy to key educational outcomes, few studies have tested whether these beliefs can be enhanced through structured university-based interventions, highlighting the need for experimental or quasi-experimental research in pre-service teacher education.

#### 1.1.3. Emotional Training in Initial Teacher Education

The growing interest in the development of socio-emotional competencies in teacher education reflects the demands of a more inclusive, empathetic, and student-centered school environment. Accordingly, universities have increasingly incorporated emotional training alongside pedagogical preparation (Abdullateef et al., 2023; Cristóvão et al., 2023; Pertegal-Felices et al., 2017). Integrating emotional education and the promotion of self-efficacy into early stages of teacher preparation acknowledges the deeply emotional nature of teaching (Savina et al., 2025). The classroom is a space where relationships, tensions, expectations, and emotions intersect, affecting both teachers and learners. In this setting, developing personal resources for emotional regulation, empathy, communication, and self-confidence becomes indispensable (Ma et al., 2023).

Psychoeducational interventions aimed at enhancing these competencies have shown positive outcomes in terms of psychological well-being, academic performance, and professional commitment. When designed using active, participatory, and reflective methodologies, they foster meaningful and lasting learning and strengthen transversal teaching skills (Frenzel et al., 2021; Gomes-Koban et al., 2019; Ma et al., 2023). However, evidence remains limited—particularly in Spanish-speaking contexts—and few studies use experimental or quasi-experimental designs (Torres-Hernández, 2025).

Recent intervention studies in pre-service teacher education support the feasibility of developing socio-emotional competencies through structured programs, although formats and evaluation approaches vary substantially. For example, Özdemir and Dilekmen (2024) implemented a 10-week social and emotional learning–focused program and reported improvements in emotional intelligence using the Bar-On EQ-i within a controlled quasi-experimental design. In contrast, Kyriazopoulou and Pappa (2023) evaluated a two-week intervention and assessed trait EI using TEIQue-SF alongside reflective diaries; although quantitative changes were not statistically significant, qualitative findings suggested benefits in emotion identification and self-awareness. Similarly, Poveda-Brotons et al. (2024) evaluated an online EI program using a quasi-experimental design with a control group and reported positive effects on academic achievement. A quasi-experimental study assessed EI using the TMMS-24 and reported gains in specific dimensions such as emotional attention and clarity (Troncoso-Ulloa et al., 2025). A recent PRISMA-based systematic review of socio-emotional intervention programs for pre-service teachers (2019–2024) highlighted the predominance of self-report measures and the lack of a uniform evaluation framework (Monroy & Manzanal, 2025). Building on this evidence, and in contrast to the heterogeneity observed across recent interventions, the present study extends prior work by integrating a multi-instrument evaluative framework within the same intervention. Specifically, OSTES captures teacher self-efficacy beliefs directly linked to teaching action, TMMS-24 assesses perceived meta-emotional processing (attention, clarity, repair), and EQ-i provides a broader profile of socio-emotional competencies related to coping and interpersonal functioning. By combining these three instruments within a single design, the study moves beyond the more common single-instrument or single-construct evaluations reported in prior interventions, enabling change to be examined across complementary facets of pre-service teachers’ professional and socio-emotional functioning.

Therefore, we provide empirical evidence from the Spanish context by evaluating an eight-week psychoeducational program through a quasi-experimental pretest–posttest design with a comparison group, testing time × group interaction effects on perceived emotional intelligence, socio-emotional competencies, and teacher self-efficacy. This responds to the need for more comparable quasi-experimental evidence in the field.

### 1.2. Teacher Training Program

The psychoeducational program implemented in this study was designed to enhance emotional intelligence and strengthen perceived teacher self-efficacy among university students enrolled in initial teacher education degrees. The training was delivered over eight consecutive weeks and focused on developing emotional awareness, regulation, empathy, and professional confidence through experiential, reflective, and practice-oriented sessions. Table 1 describes the details of each lesion in the program.

### 1.3. Objectives and Hypotheses

The general objective of this study is to evaluate the effectiveness of a psychoeducational intervention program in enhancing emotional intelligence and perceived teacher self-efficacy among university students enrolled in initial teacher education.

In line with this objective, the following specific objectives and working hypotheses are proposed:

Specific Objective 1: Analyze pre-post changes in the experimental and control groups after the implementation of the intervention, in teacher self-efficacy (OSTES), perceived emotional intelligence (TMMS-24), and socio-emotional competencies (EQ-i).

Specific Objective 2: Examine dimension-level patterns of change across OSTES, TMMS-24, and EQ-i in relation to the intervention content.

The general hypothesis would be formulated as follows:

There are significant improvements in perceived teacher self-efficacy, emotional intelligence, and socio-emotional competencies among university students who participate in the intervention program, compared to those in the control group.

**Hypothesis** **1** (overall effect)**:** *The experimental group will show greater pre-post improvements than the control group across OSTES, TMMS-24, EQ-i outcomes.*

Theoretical rationale: Targeted training experiences and self-regulatory learning opportunities may strengthen efficacy beliefs and emotion-related functioning (Bandura, 1997), and emotional intelligence frameworks emphasize skills related to understanding and managing emotions that are expected to be sensitive to structured learning contexts (Mayer & Salovey, 1997).

**Hypothesis** **1a** (OSTES)**:** *The experimental group will show greater gains than the control group in instructional strategies, classroom management, and student engagement.*

Theoretical rationale: Teaching self-efficacy reflects beliefs about one’s capability to organize and execute teaching-related actions and can be strengthened through guided practice, feedback, and self-regulatory processes (Bandura, 1997; Tschannen-Moran & Hoy, 2001).

**Hypothesis** **1b** (TMMS-24)**:** *The experimental group will exhibit significantly greater post-intervention improvements than the control group in emotional clarity and emotional repair. Changes in emotional attention will be examined exploratorily, given that attention may be more dependent on individual monitoring style and on whether intervention activities explicitly target emotional monitoring.*

Theoretical rationale: TMMS-24 assesses perceived meta-emotional processes (attention, clarity, repair), and training activities that enhance emotional awareness, understanding, and regulation are expected to improve these perceived processes (Mayer & Salovey, 1997; Extremera Pacheco & Fernández-Berrocal, 2004).

**Hypothesis** **1c** (EQ-i)**:** *The experimental group will exhibit significantly greater post-intervention improvements than the control group across EQ-i dimensions (intrapersonal, interpersonal, stress management, adaptability, and general mood, as well as the total score).*

Theoretical rationale: The EQ-i framework conceptualizes emotional-social intelligence and a set of competencies related to coping, adaptability, and socio-emotional functioning, which may be strengthened through psychoeducational training targeting socio-emotional learning and regulation (Bar-On, 1997, 2006).

## 2. Materials and Methods

### 2.1. Participants

A total of 163 university students enrolled in the bachelor’s degrees in early childhood and Primary Education at a Spanish public university participated in this study. The experimental group (*n* = 90) received a psychoeducational intervention, while the control group (*n* = 73) continued with their regular academic activities.

The mean age of the participants was 18.66 years (SD = 2.38), with an age range between 17 and 48 years old. Of the total sample, 89.6% were women and 10.4% were men.

Inclusion criteria were: (a) being enrolled in one of the degrees, (b) not having received prior training in emotional education, and (c) voluntarily agreeing to participate in the study through informed consent. The sampling procedure was non-probabilistic and based on convenience, typical of real educational settings. Although no information was collected regarding participants’ socioeconomic status, a relatively homogeneous profile is assumed given their attendance at a public university.

To ensure that the changes observed were exclusively due to the intervention, conditions were kept consistent between the experimental group (EG) and the control group (CG). Specifically, in terms of curriculum, both groups took the same courses and followed the same official programs approved by the university. In terms of teaching, the teachers in both groups were the same and were coordinated to ensure the development of the same personal and professional competencies established for early childhood and primary school teachers. In terms of context, the participants in both groups belonged to the same Faculty of Education.

### 2.2. Instruments

The instruments employed in this study are described below, in line with the study objectives and hypotheses.

1. Teachers’ Sense of Efficacy Scale (OSTES): Developed by Tschannen-Moran and Hoy (2001), the OSTES evaluates perceived teacher self-efficacy across three core dimensions: Instructional Strategies (e.g., “How well can you implement alternative instructional strategies in your classroom?”), Classroom Management (e.g., “How much can you do to control disruptive behavior in the classroom?”), and Student Engagement (e.g., “How much can you do to motivate students who show low interest in schoolwork?”)

In prior Spanish validations, the OSTES has shown good internal consistency, with Cronbach’s alpha coefficients of 0.78 for Instructional Strategies, 0.85 for Classroom Management, 0.82 for Student Engagement, and 0.91 for the total scale (Salas-Rodríguez et al., 2021).

2. Trait Meta-Mood Scale-24 (TMMS-24): The TMMS-24 is a self-report questionnaire originally developed by Salovey et al. (1995) and later adapted and validated for the Spanish population by Fernández-Berrocal et al. (2004). This instrument measured perceived emotional intelligence based on Salovey and Mayer’s ability model. It evaluates three dimensions: Emotional Attention (e.g., “I pay a lot of attention to how I feel”), Emotional Clarity (e.g., “I almost always know exactly how I feel”), and Emotional Repair (e.g., “Although I sometimes feel sad, I usually have an optimistic outlook”). The scale consists of 24 items, rated on a 5-point Likert scale from 1 (Strongly disagree) to 5 (Strongly agree).
  In university samples, the TMMS-24 has demonstrated high internal consistency, with Cronbach’s alpha values of 0.90 for Attention, 0.90 for Clarity, and 0.86 for Repair (Extremera Pacheco & Fernández-Berrocal, 2004).
3. Emotional Quotient Inventory (EQ-i): The EQ-i, developed by Bar-On (1997), measures trait emotional intelligence as a broader personality-related construct. The version used 30 items covering five dimensions: Intrapersonal (e.g., emotional self-awareness, independence), Interpersonal (e.g., empathy, interpersonal relationships), Adaptability (e.g., problem solving, flexibility), Stress Management (e.g., stress tolerance, impulse control), and General Mood (e.g., optimism, happiness). Responses were given on a 4-point Likert scale ranging from 1 (Not true of me) to 4 (Very true of me). The EQ-i has shown strong psychometric properties, with Cronbach’s alpha coefficients ranging from 0.86 to 0.97 across its subscales (Bharwaney et al., 2011).

### 2.3. Procedure

The intervention started in February 2024, during the first semester of the academic year. Prior to implementation, institutional approval was obtained, and participants were informed of the study’s objectives, structure, and voluntary nature. All participants provided informed consent.

The selection of groups followed a naturalistic criterion, as the students assigned to the experimental and control groups were already enrolled in existing university courses led by the principal investigator. This approach allowed for the program to be implemented within the real conditions of teacher education, aligning with the ecological validity of the study.

The experimental group attended a psychoeducational program lasting eight weeks, with weekly one-hour face-to-face sessions, facilitated by the principal investigator, a psychologist specialized in educational psychology and teacher training.

Each session focused on a specific thematic axis, incorporating structured activities designed to help students connect emotional content with real-world educational scenarios. The first sessions focused on emotional self-awareness and identification of one’s emotional responses. Subsequent sessions addressed emotional comprehension and the application of regulation strategies and emotional reframing. Midway through the program, sessions focused on classroom emotional dynamics, management of emotionally charged situations, and peer collaboration. In the final weeks, the development of empathy, conflict resolution skills, and teacher self-efficacy beliefs were reinforced through role-plays, video analysis, and guided self-reflection. The final session included the administration of the posttest instruments and a collective reflective activity.

Methodologies used were active and reflective, including group dynamics, role-playing, case study analysis, emotion regulation techniques, and self-exploration exercises. Each session concluded with a brief relaxation or grounding activity and a voluntary space for sharing experiences, aimed at promoting emotional integration and peer support. 

The instruments were administered in digital format via Google Forms at two time points, before (pretest) and after (posttest) the intervention. Data confidentiality and voluntary participation were ensured throughout the study.

Between the pretest and posttest phases, an attrition rate of 19.3% (*n* = 39) was recorded, mainly due to absence from the final sessions or incomplete posttest responses. This dropout rate did not exceed 20% of the initial sample and was therefore considered acceptable for the purposes of statistical analysis.

### 2.4. Experimental Design and Data Analysis

A quasi-experimental design with a non-equivalent control group and repeated measures (pretest and posttest) was employed. This design was selected due to the natural constraints of the university context, where random assignment was not feasible due to ethical and logistical considerations, such as academic scheduling and class structure.

Although true experimental designs provide stronger causal inference, quasi-experimental designs with control groups and repeated measures increase internal validity by allowing longitudinal comparisons between groups (Shadish et al., 2002). Nonetheless, the absence of randomization may introduce selection biases and should be considered when interpreting results. The design used, a pretest-posttest design, allows for the measurement of gain scores (improvement) rather than just final results. In addition, it allows for the identification of potential threats to internal validity, such as selection x maturation interaction or differential statistical regression, depending on the pattern of actual results obtained.

Data were analyzed using SPSS software (version 27.0), licensed by the University of Alicante (Spain). The analyses included descriptive statistics, reliability analyses, and general linear models (GLM) for repeated measures, in order to assess both main and interaction effects (time × group) on the study variables.

All procedures were conducted in accordance with ethical standards and approved by the Ethics Committee. The study was initially covered by the approval of 22 December 2021 (Approval Code: UA-2021-12-09_2) and was subsequently included in the project continuation approved on 29 September 2025 (Approval Code: UA-2025-09-22_1). Both approvals ensured compliance with applicable ethical guidelines, including informed consent, confidentiality, and voluntary participation.

Generative AI was used only for translation (Spanish to English) and language editing. No GenAI tools were used to generate scientific content, analyze data, interpret results, or develop study protocols.

## 3. Results

Initially, we examined whether there were statistically significant differences between the experimental and control groups across the variables assessed in the study prior to the intervention. For this purpose, an independent samples *t*-test was conducted. As presented in Table 2, significant differences were observed in the variables Classroom Management (involved in the OSTES), Intrapersonal Intelligence and Stress Management (involved in the EQ-i), and the EQ-i total score, with the control group displaying higher mean scores across all four dimensions. No statistically significant differences were found in Instructional Strategies and Student Engagement (involved in the OSTES), Attention, Clarity, and Repair (involved in the TMMS), as well as Interpersonal Intelligence, Adaptation, and General Mood (involved in the EQ-i), suggesting a general equivalence between groups at baseline.

Secondly, Box’s M test was conducted to examine whether the assumption of homogeneity of variance-covariance matrices was supported for the variables included in the OSTES, TMMS-24, and EQ-i questionnaires. The test indicated homogeneity of the variance-covariance matrices for Student Engagement (involved in the OSTES) (F = 1.64; df = 11,010,653.50; *p* = .177).

However, the test results indicated non-homogeneity in the following variables involved in the OSTES: Instructional Strategies (F = 6.81; df = 11,010,653.50; *p* = .000) and Classroom Management (F = 4.38; df = 11,010,653.50; *p* = .004). Similarly, non-homogeneity was also observed for all dimensions assessed with the TMMS-24, including Attention (F = 4.061; df = 11,010,653.50; *p* = .007), Emotional Clarity (F = 13.76; df = 11,010,653.50; *p* = .000), and Emotional Repair (F = 11.64; df = 11,010,653.50; *p* = .000). Regarding the EQ-i, the Box’s M test suggested non-homogeneity for Intrapersonal Intelligence (F = 17.71; df = 11,010,653.50; *p* = .000), Interpersonal Intelligence (F = 4.23; df = 11,010,653.50; *p* = .005), Stress Management (F = 22.759; df = 11,010,653.50; *p* = .000), Adaptability (F = 10.652; df = 11,010,653.50; *p* = .000), General Mood (F = 13.58; df = 11,010,653.50; *p* = .000), as well as for the EQ-i Total Score (F = 21.621; df = 11,010,653.50; *p* = .000). Nevertheless, it should be remembered that violations of this assumption are considered to have a limited impact when group sizes are approximately equal (Hair et al., 1999; Field, 2013). While Box’s M test indicated a violation of homogeneity of covariance matrices (*p* < .05), the analysis proceeded using a Brown-Forsythe F adjustment for univariate follow-ups, which is robust to such violations.

Finally, the results derived from the analysis of intra-subject and inter-subject effects are presented in Table 3. These results reveal that the interaction effect between the evaluation time (pre- and post-test) and group condition (experimental vs. control) was statistically significant (*p* < .05) for the participants in the experimental group compared to the control group, showing improvements in the following dimensions:

1. A significant improvement in teachers’ self-efficacy was observed:
  1.1 Confirmed through the OSTES, with statistically significant interaction effects identified in the dimensions of Instructional Strategies and Classroom Management. These results indicate a greater enhancement over time in the experimental group compared to the control group.
  1.2 Although a statistically significant interaction effect was also observed for Student Engagement, the associated effect size was in the medium range (ηp^2^ = 0.13), and the result should be interpreted in light of the overall pattern of findings.
2. A significant improvement in emotional intelligence was found:
  2.1 Confirmed through the TMMS-24, with significant interaction effects in the dimensions of Emotional Clarity and Emotional Repair.
  2.2 No significant interaction effect was found for Attention, suggesting that the changes observed in this dimension may not be attributable to the intervention.
3. A significant improvement in both global and specific emotional competencies was observed:
  3.1 Confirmed through the EQ-i, with statistically significant interaction effects observed in the dimensions of Intrapersonal Intelligence, Interpersonal Intelligence, Stress Management, Adaptability, General Mood, and in the EQ-i Total Score.

These findings reflect significant post-intervention gains in emotional competencies among participants in the experimental group.

**Figure 1:**
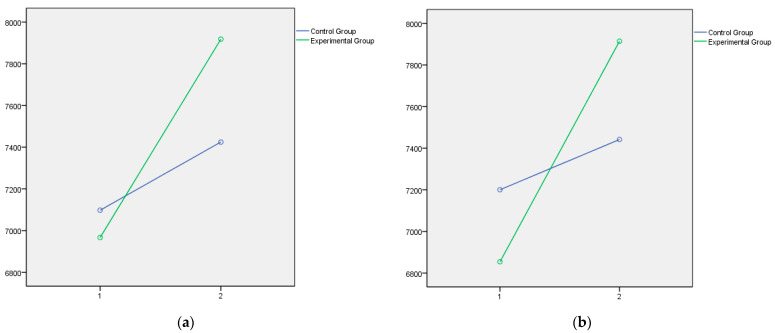
(**a**) Efficacy in Instructional Strategies after the educational intervention measures with the Teachers’ Sense of Efficacy Scale is contained in the first panel; (**b**) Efficacy in Classroom Management after the educational intervention measures with the Teachers’ Sense of Efficacy Scale Questionnaire is contained in the second panel.

**Figure 2:**
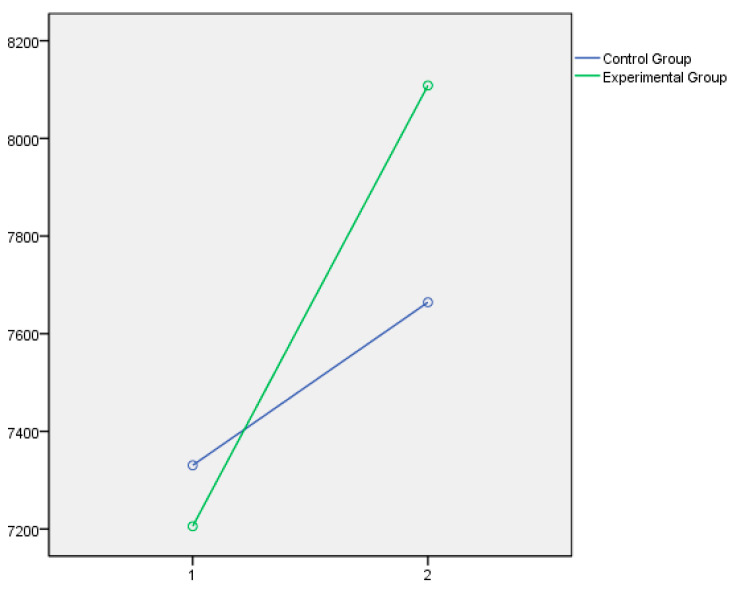
Efficacy in Student Engagement after the educational intervention measures with the Teachers’ Sense of Efficacy Scale Questionnaire.

**Figure 3:**
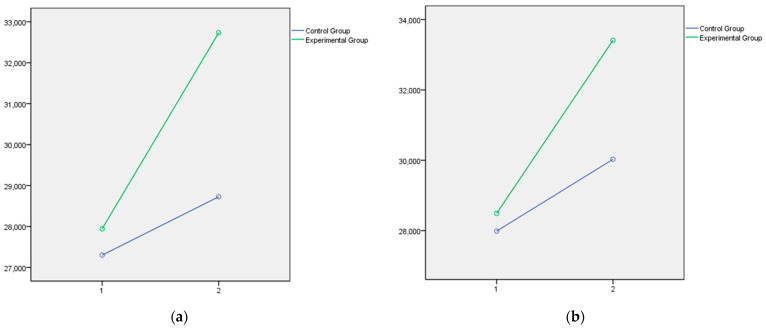
(**a**) Clarity after the educational intervention measures with Trait Meta-Mood Scale is contained in the first panel; (**b**) Repair after the educational intervention measures with Trait Meta-Mood Scale is contained in the second panel.

**Figure 4:**
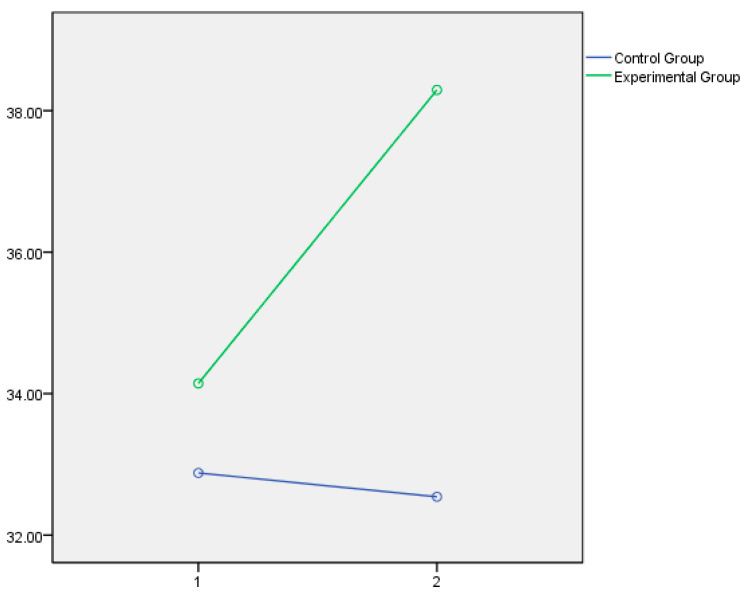
EQ-i Total Score as a representation of the significant differences obtained in the set of factors of EI measures with Emotional Quotient Inventory.

Lastly, the graphical representation of the variables that showed statistically significant differences between the experimental and control groups is presented below. In this respect, Figure 1 and Figure 2 illustrate the dimensions of Instructional Strategies, Classroom Management, and Student Engagement, respectively, as measured by the Teachers’ Sense of Efficacy Scale (OSTES). The graphs reflect the distinct effects of the intervention on each of these components. Figure 3 displays the emotional intelligence dimensions of Emotional Clarity and Emotional Repair measured with the TMMS-24, reflecting the significant changes observed following the educational program. Lastly, Figure 4 presents the EQ-i Total Score as a global indicator of emotional intelligence, summarizing the significant differences obtained across the set of factors assessed with the EQ-i.

In all cases, the direction of the significant effects indicates a clear pattern of improvement in the experimental group following the intervention.

Furthermore, the actual pattern of results, with the experimental group starting from a lower value than the control group, makes it unlikely that there is a threat to internal validity due to selection x maturation, that is, that the experimental group improves because it starts with a higher score rather than because of the effect of the treatment, in all cases except for the total EQ-i emotional intelligence score, where the difference in the pretest between the experimental and control groups is marginally significant in favor of the experimental group. However, even in this case, the magnitude of the effect size (0.38) also makes a threat to internal validity of this type unlikely, and the change in EQ-i can most likely be attributed to the effect of the intervention.

In summary, the psychoeducational intervention had significant effects on the three factors of teacher efficacy, which in all cases can be considered moderate in size. The intervention program accounted for 15% of the variance in efficacy in instructional strategies, 19% in efficacy in classroom management, and 13% in efficacy in student engagement. The effect of the program is more moderate on the emotional intelligence variables assessed by the TMMS, with no significant effects on emotional attention and small effects on emotional clarity (7%) and emotional repair (6%). However, the effects on the emotional intelligence variables assessed by the EQ-i were large in all cases, ranging from 15% on interpersonal intelligence and stress management, to 28% on intrapersonal intelligence, with a notable effect on the Total EQ-i, which reached 38%.

## 4. Discussion

The findings of this study provide consistent evidence of the effectiveness of a structured psychoeducational intervention aimed at enhancing teachers’ emotional intelligence, self-efficacy, and socio-emotional competencies. The assessment of these constructs through validated instruments, OSTES, TMMS-24, and EQ-i, allowed for a comprehensive analysis of the program’s impact across intrapersonal and professional domains.

According to the results obtained from the Ohio State Teacher Efficacy Scale (OSTES), the intervention yielded significant improvements in instructional strategies, classroom management, and student engagement. These findings align with previous research linking emotional intelligence with pedagogical effectiveness (de Ruig et al., 2024; De Smul et al., 2018). Teachers in the experimental group demonstrated greater capacity to adapt their instructional practices, suggesting that enhanced emotional competencies may contribute to more effective lesson planning and delivery. In classroom management, significant gains were also observed, reinforcing the idea that emotional regulation is key to fostering a positive learning environment and managing student behavior (Pattiasina et al., 2024). Furthermore, although the effect size for student engagement was modest, the interaction effect was statistically significant, indicating a positive influence of the program on teachers’ perceived ability to motivate and involve students actively in the learning process (Savina et al., 2025). The lack of significant between-group effects when analyzed independently further highlights the importance of considering the interaction between group and time, which consistently favored the experimental group.

Regarding perceived emotional intelligence, assessed using the Trait Meta-Mood Scale (TMMS-24), a differential and progressive impact of the intervention was noted with significant gains in emotional clarity and repair in the experimental group, while attention remained stable. This pattern is plausible because TMMS-24 attention reflects emotion monitoring, which may be less responsive to short-term training and more dependent on individual monitoring style. Moreover, increased attention is not uniformly adaptive if it is not accompanied by sufficient clarity and regulation (it may foster rumination). From a practical perspective, the program components were designed primarily to strengthen emotion understanding and regulation strategies, which align more directly with clarity and repair than with increasing emotional monitoring.

This finding is consistent with the theoretical framework by Mayer and Salovey, which suggests a hierarchical development of emotional competencies. Emotional clarity, which allows individuals to understand and differentiate emotions, showed meaningful gains, suggesting that the intervention fostered deeper emotional comprehension beyond the potential effects of instrument repetition (Castillo et al., 2013). Similarly, the enhancement in emotional repair reflects improved abilities to manage negative affect and restore emotional balance, as supported by recent studies on emotional regulation (Iovino et al., 2021; Mitsea et al., 2024; Tang et al., 2019). In terms of convergence with prior intervention research in initial teacher education, changes in TMMS-24 dimensions may depend on program emphasis and format. For instance, Troncoso-Ulloa et al. (2025) reported significant gains in emotional attention and clarity, whereas our program emphasized understanding and regulation strategies, which may help explain improvements in clarity and repair while attention remained stable. Although attention did not register significant interaction effects, its stability in the experimental group—contrasted with a decline in the control group—may indicate a protective effect of the program against emotional disengagement. These findings reinforce the importance of designing emotional education programs that go beyond awareness to include active emotional regulation strategies. From a data-based standpoint, the intervention was associated with differential gains in clarity and repair (Time × Group), whereas the interpretation of why attention remained stable is considered exploratory and warrants direct testing in future research.

The outcomes derived from the Emotional Quotient Inventory (EQ-i) further support the efficacy of the intervention in promoting a broad range of socio-emotional competencies. Significant interaction effects were observed for all dimensions evaluated, including intrapersonal and interpersonal intelligence, stress management, adaptability, general mood, and the global EQ-i score. Participants in the experimental group exhibited enhanced flexibility and coping abilities, particularly in stress management and adaptability, core skills for navigating the emotional demands of the teaching profession (Chen et al., 2025; Ghasemi et al., 2024). The dimension of general mood also improved, with greater response homogeneity, suggesting the internalization of optimism and emotional resilience, consistent with literature linking general mood to adaptive stress responses (Extremera & Fernández-Berrocal, 2005). The improvement in interpersonal skills was equally noteworthy, reflecting a strengthened ability to engage empathetically and effectively in social interactions, a key competence for establishing supportive classroom climates. Lastly, the significant gain in the overall EQ-i score confirms the wide-reaching impact of the program on emotional functioning.

Importantly, the present findings should not be interpreted as unique to this specific program. Rather, the observed improvements are likely driven by transferable structural elements: a sequenced progression from awareness to understanding and regulation, participatory and experiential methodologies (case-based analysis, role-playing with guided feedback, and structured group reflection), and repeated opportunities to practice and consolidate skills across sessions.

In practice, teacher educators and mentors can implement brief weekly sessions combining an emotionally demanding case, a guided role-play with feedback, and a brief reflection task to support transfer to classroom decision-making. Simple emotion regulation routines (e.g., reframing prompts, coping planning) may be especially useful for strengthening clarity and repair, and brief emotion-labeling check-ins could be added to foster adaptive attention.

Despite these promising results, several limitations must be acknowledged. First, the quasi-experimental design, without random assignment, poses a risk of self-selection bias. It is possible that participants in the experimental group were more intrinsically motivated or predisposed to emotional development. This design feature also increases the likelihood of baseline nonequivalence between groups, as reflected in the small pretest differences observed in a subset of variables. Second, although pretest differences were minimal for most variables, some disparities in intrapersonal intelligence, stress management, and classroom management are plausible in quasi-experimental studies using intact classrooms and likely reflect pre-existing cohort characteristics rather than intervention effects. These baseline imbalances imply that between-group differences at posttest should be interpreted cautiously, prioritizing differential change over time (Time × Group) rather than absolute end-point comparisons. Although no covariate-adjusted analyses were conducted in the present study, the consistency of the Time × Group pattern across most outcomes reduces the likelihood that the findings are driven solely by initial nonequivalence. Nevertheless, future studies should replicate these results using random assignment and/or covariate-adjusted models (e.g., ANCOVA or mixed-effects approaches) to further strengthen causal inference. Third, the relatively short intervention period (eight weeks) and modest sample size may constrain the generalizability of the findings. Furthermore, reliance on self-report instruments introduces the potential for social desirability bias and common-method variance, as responses may reflect participants’ self-perceptions, expectations, or increased awareness rather than observable behavioral change. This is particularly relevant for constructs such as emotional intelligence and self-efficacy, where perceived improvement may not fully correspond to enacted competencies in authentic teaching situations. Future research should therefore triangulate outcomes using complementary data sources, such as observational indicators during practicum or simulated teaching tasks, external ratings from mentors/supervisors, and performance-based measures of emotional skills. Incorporating qualitative methods (e.g., reflective journals or interviews) and social desirability controls would further strengthen validity and clarify whether changes generalize beyond self-reported gains. While the quasi-experimental nature of this study—utilizing intact classrooms—limits the ability to control for all potential confounding variables, the use of the design allows change over time to be examined, helping to identify patterns consistent with intervention-related gains.

Future research should aim to address these limitations by incorporating larger and more diverse samples, employing randomized controlled designs, and including behavioral or observational measures to complement self-report data. Longitudinal follow-ups are also recommended to evaluate the sustainability of the observed improvements over time. Additionally, exploring potential moderators (e.g., gender, academic motivation, institutional climate) may yield insights into for whom and under what conditions the intervention is most effective.

These findings also align with prior research demonstrating that multimodal training formats can enhance both emotional competencies and professional preparedness. For instance, Pozo-Rico et al. (2018) implemented a blended program combining face-to-face sessions, online learning, and personalized coaching, which significantly improved emotional intelligence in pre-service teachers. Moreover, the intervention strengthened transversal competencies such as communication, teamwork, and time management—skills essential for professional success in educational contexts. This reinforces the idea that emotionally focused interventions have a ripple effect, positively influencing both intrapersonal development and pedagogical efficacy.

In summary, the results underscore the functional value of implementing emotionally focused psychoeducational interventions within initial teacher training. These programs not only enhance emotional intelligence and self-efficacy but also contribute to the development of critical socio-emotional skills essential for effective teaching. As such, higher education institutions are encouraged to integrate emotional education modules or workshops into teacher preparation curricula. Doing so may better equip future educators to navigate the emotional complexity of classroom environments, ultimately promoting both teacher well-being and student outcomes. These conclusions are consistent with recent research advocating for the integration of social-emotional learning in teacher education as a foundation for holistic educational development (Moreira-Choez et al., 2024).

## 5. Conclusions

This study suggests that an eight-week psychoeducational intervention grounded in emotional intelligence is associated with improvements in pre-service teachers’ self-efficacy, socio-emotional competencies, and perceived emotional intelligence, relative to the stability observed in the control group. However, given the quasi-experimental design, these results should be interpreted as evidence of differential change between groups rather than definitive causal effects.

From a practical perspective, the findings support integrating brief socio-emotional modules into initial teacher education, especially applied and reflective activities (e.g., case-based analysis, role-plays with guided feedback, and structured self-reflection) combined with explicit training in emotion regulation strategies and cognitive reframing. Because emotional attention did not show a significant change, future iterations could incorporate brief tasks that promote adaptive emotion monitoring (e.g., short check-ins and emotion-labeling tasks) alongside clarity- and regulation-focused components.

Future research should strengthen inference and external validity by using randomized or cluster-randomized designs, when feasible, incorporating longitudinal follow-ups (e.g., during practicum placements), and complementing self-report data with observational or performance-based measures (e.g., simulations or external ratings).

Overall, this study provides relevant empirical evidence supporting the integration of emotional education into initial teacher training. The findings suggest that such programs may foster a more inclusive, resilient, and effective teaching practice. Ensuring the long-term sustainability of these interventions will require addressing institutional barriers, such as curricular overload and resource availability.

## Figures and Tables

**Table 1:** Training program for pre-service teachers (8-week psychoeducational intervention).

	Lesson	Target	Tasks
1	‘Pretest and Emotional Awareness’	- Administer pretest instruments (OSTES, TMMS-24, EQ-i). - Promote recognition and vocabulary of emotions. - Facilitate initial self-reflection.	Pretest questionnaire administration. Group dynamics: “Name your emotion” Brainstorm: What do teachers feel? EI introductory reflection questions.
2	‘Understanding Emotions’	- Deepen awareness of emotional triggers. - Recognize emotional responses in educational settings.	Emotion mapping. Group discussion on emotional triggers. “When I feel…” reflective worksheet.
3	‘Emotion regulation I’	- Develop self-regulation strategies. - Identify personal stressors in teaching. - Practice basic regulation techniques.	“My stress curve” timeline. Breathing and grounding practice. Group sharing.
4	‘Emotion regulation II’	- Apply regulation in complex situations. - Introduce techniques for real-time classroom regulation. - Reinforce self-care habits.	Case study: Classroom conflict management. Role-play: managing emotional triggers. Relaxation practice.
5	‘Teachers self-efficacy I’	- Explore beliefs about teaching competence. - Identify personal strengths and challenges.	“My teaching mirror” self-reflection. Group discussion on teacher impact. Visual metaphor drawing.
6	‘Teachers self-efficacy II’	- Practice classroom management strategies. - Strengthen instructional self-efficacy.	Microteaching simulations. Peer feedback circle. “Difficult classroom” video analysis.
7	‘Empathy and relationships’	- Foster empathetic communication. - Address conflict resolution through emotional perspective-taking.	“In your shoes” role play. Active listening techniques. Reflective writing: “Understanding my student.”
8	‘Integration and closure’	- Integrate learning across sessions. - Reflect on emotional and professional growth. - Administer posttest instruments.	Posttest questionnaire administration. “Letter to my future teacher self.” Group closing circle.

**Table 2:** Teacher’s *t*-test results for the difference in mean scores (before training).

Variables	t	df	Sig.	Difference	SD
Instructional Strategies	1.11	199.00	0.27	0.14	0.13
Classroom Management	3.02	199.00	0.00	0.38	0.13
Student Engagement	1.70	199.00	0.09	0.21	0.13
Attention	−1.12	199.00	0.27	−0.84	0.75
Clarity	−0.96	199.00	0.34	−0.84	0.88
Repair	0.19	199.00	0.85	0.15	0.83
Intrapersonal intelligence	−2.23	199.00	0.03	−1.87	0.84
Interpersonal intelligence	−0.15	199.00	0.88	−0.09	0.59
Stress management	−2.29	199.00	0.02	−2.10	0.92
Adaptation	−1.24	199.00	0.22	−0.77	0.63
General Mood	−0.30	199.00	0.76	−0.24	0.79
EQ-i total	−1.95	199.00	0.05	−1.02	0.52

**Table 3:** Results of intrasubject/intersubject univariate analysis of variance (ANOVA).

Area Examined	Effect	F(1,161)	*p*	η^2^
Efficacy in Instructional Strategies	Time	124.03	<.001	0.43
	Time × Group	29.58	<.001	0.15
	Group	2.43	.120	0.01
Efficacy in Classroom Management	Time	98.49	<.001	0.38
	Time × Group	38.95	<.001	0.19
	Group	0.30	.580	0.00
Efficacy in Student Engagement	Time	119.13	.420	0.00
	Time × Group	25.21	<.001	0.13
	Group	2.21	.140	0.01
Attention	Time	6.50	.010	0.04
	Time × Group	2.01	.158	0.01
	Group	9.05	<.001	0.05
Clarity	Time	40.30	<.001	0.20
	Time × Group	11.81	<.001	0.07
	Group	9.51	<.001	0.06
Repair	Time	61.44	<.001	0.28
	Time × Group	10.52	<.001	0.06
	Group	7.92	.0100	0.05
Intrapersonal intelligence	Time	37.45	<.001	0.19
	Time × Group	63.48	<.001	0.28
	Group	51.30	<.001	0.24
Interpersonal intelligence	Time	4.26	.040	0.02
	Time × Group	29.10	<.001	0.15
	Group	12.15	<.001	0.07
Stress management	Time	41.35	<.001	0.20
	Time × Group	28.76	<.001	0.15
	Group	29.87	<.001	0.16
Adaptation	Time	45.04	<.001	0.22
	Time × Group	30.47	<.001	0.16
	Group	24.58	<.001	0.13
General Mood	Time	24.69	<.001	0.13
	Time × Group	46.79	<.001	0.22
	Group	17.44	<.001	0.09
EQ-i Total Score	Time	72.45	<.001	0.31
	Time × Group	100.34	<.001	0.38
	Group	51.92	<.001	0.24

Note. Sig. level *p* ≤ .05. Time = within-subjects effect (pretest–posttest); Group = between-subjects effect (experimental vs. control); Time × Group = interaction effect.

## Data Availability

The raw data supporting the conclusions of this article will be made available by the authors on request.
